# Acoustic Emission from Porous Collapse and Moving Dislocations in Granular Mg-Ho Alloys under Compression and Tension

**DOI:** 10.1038/s41598-018-37604-5

**Published:** 2019-02-04

**Authors:** Yan Chen, Xiangdong Ding, Daqing Fang, Jun Sun, Ekhard K. H. Salje

**Affiliations:** 1 State Key Laboratory for Mechanical Behavior of Materials, Xi’an Jiaotong University, Xi’an, 710049 China; 2 Department of Earth Sciences, University of Cambridge, Cambridge, CB2 3EQ England

## Abstract

We identified heterogeneous Mg-Ho alloys as an ideal material to measure the most extensive acoustic emission spectra available. Mg-Ho alloys are porous and show a high density of dislocations, which slide under external tension and compression. These dislocations nucleate near numerous heterogeneities. Two mechanisms compete under external forcing in the structural collapse, namely collapsing holes and the movements of dislocations. Their respective fingerprints in acoustic emission (AE) measurements are very different and relate to their individual signal strengths. Porous collapse generates very strong AE signals while dislocation movements create more but weaker AE signals. This allows the separation of the two processes even though they almost always coincide temporarily. The porous collapse follows approximately mean-field behavior (ε = 1.4, τ’ = 1.82, α = 2.56, *x* = 1.93, χ = 1.95) with mean field scaling fulfilled. The exponents for dislocation movement are greater (ε = 1.92, τ’ = 2.44, α = 3.0, *x* = 1.7, χ = 1.42) and follows approximately the force integrated mean-field predictions. The Omori scaling is similar for both mechanisms. The Bath’s law is well fulfilled for the porous collapse but not for the dislocation movements. We suggest that such ‘complex’ mixing behavior is dominant in many other complex materials such as (multi-) ferroics, entropic alloys and porous ferroelastics, and, potentially, homogeneous materials with the simultaneous appearance of different collapse mechanisms.

## Introduction

When materials are compressed or extended, they often emit ‘crackling noise’. These are sharp sound signals, which stem from rapid structural changes inside the material. Such sound signals, also called acoustic jerks, are an extremely widespread phenomenon and can sometimes be measured with commercial sound detection systems. We explore whether such sound signals are characteristic for specific structural changes such as the nucleation of twin boundaries, the movement of dislocations, the collapse of cavities, and the propagation of cracks^1^. More generally, jerk sequences may relate to avalanches and their evolution in space and time is a hotly debated topic in material sciences^1–19^.

Avalanches in deformed granular materials, sand piles, debris flow^2^, granular mixtures^3^, fine powders^4^, plastic flow^5^, and free surface flow^6^ are subject to intense research due to their importance in industrial processes, geophysics and engineering. Likewise, the investigation of porous materials has been in the limelight because its close similarity to Earth Quakes^7^ and can, in some cases, predict catastrophic events in the mining industry and plays a major role for structural materials, such as fillers for aircraft wings and crash-absorbers in cars^8–14^. A multitude of experimental observations are compiled in textbooks^15^. The available data demonstrate that a common shortcoming of AE spectroscopy is that the number of AE signals (~jerks) is usually too small or too weak to generate large enough data sets, which can not be analysed to identify the underlying physical process. This observation becomes even more important when several mechanisms occur simultaneously so that even the power law distributions of AE energies becomes obscured^16^.

Avalanches in complex materials with superimposed mechanisms generally show deviations from power-law distributed acoustic emission (AE) because AE contains information about all processes involved in the deformation process. They produce complex AE spectra and contain different avalanche exponents. This is not the case in standard mean-field (MF) approaches where the same mean-field exponents are expected for all processes^17^ and their superposition equally follows MF. For example, mechanical avalanches during compression of martensitic porous Ti-Ni generate two sequences of AE signals. These sequences were very difficult to disentangle because the number and intensity of AE events is very limited^18^.

We developed a strategy to design a model material where two strong AE active mechanisms are easily measurable. The previously most extensive data set is related to the collapse of the very brittle nano-porous vycor, a SiO_2_ based material, with >30000 AE events^7,8^. Other compressive experiments equally focused on brittle porous materials (e.g.,Vycor, Gelsil, sandstone, shale, calcareous schist and charcoal)^9–13^ with strong AE signals. The results showed that avalanches in brittle porous materials do indeed emit strong AE signals although their total activity is sometimes small^9,18^. We now added to porosity the most common microstructure leading to acoustic emission, namely dislocation movements. In order to optimize the number of dislocations, we choose highly heterogeneous Mg-Ho alloys, which contain multiple grains ranging from pure Ho to a maximum Mg content of 88%. High dislocation densities are related to their ease to nucleation. Dislocation tangles appear near heterogeneities, such as Ho inclusions and near grain boundaries. These dislocations render our materials slightly ductile. As a result, the characteristic AE signals of granular Mg-Ho samples include both very strong AE events from porous collapse and weak AE events from dislocation motion. We report in this paper that we could indeed observe more than 2 million AE events in Mg-Ho and separate the two main components.

In optimized Mg-Ho alloys the two competing mechanisms coexist under tension and compression. One mechanism is related to the collapse of pores, the other reflects the movement of dislocations. The corresponding AE signals are very strong for the porous collapse and weak for dislocation movements. The heterogeneous microstructure in Mg-Ho allows nucleation and the movement of dislocations during the deformation process, leading to high AE activity. This is the first experimental observation of such mix of avalanche processes with different signal strengths of the observable. We find that each sequence remains scale invariant over large intervals despite the superposition by a second sequence with different characteristics.

## Results

### Stress-strain curves and jerk spectra under tension and compression

**Figure 1:**
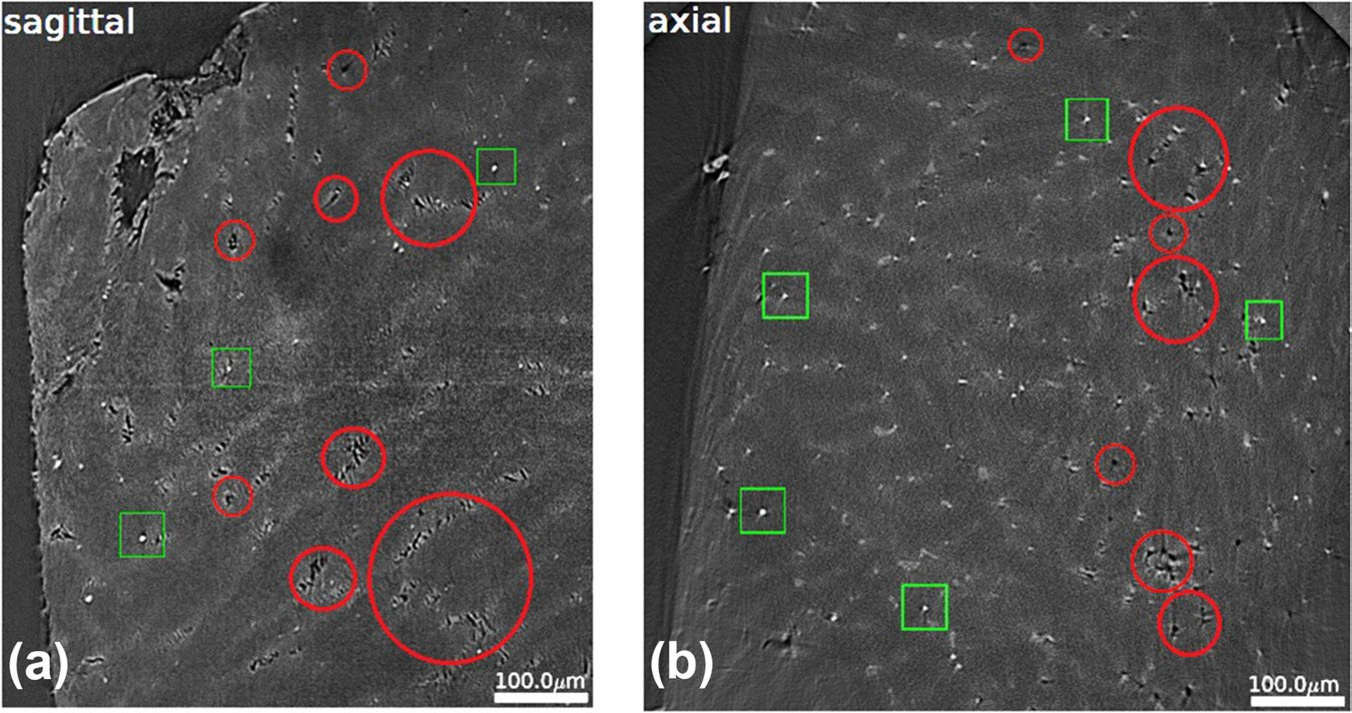
Microstructure of Mg-Ho granular porous alloys. (**a**) Sagittal section and (**b**) axial section. SEM figure shows distribution of pores (black areas, some typical holes are indicated by red circles). Simultaneously we find precipitates of Ho as white spots (some typical areas are indicated by green squares).

**Figure 2:**
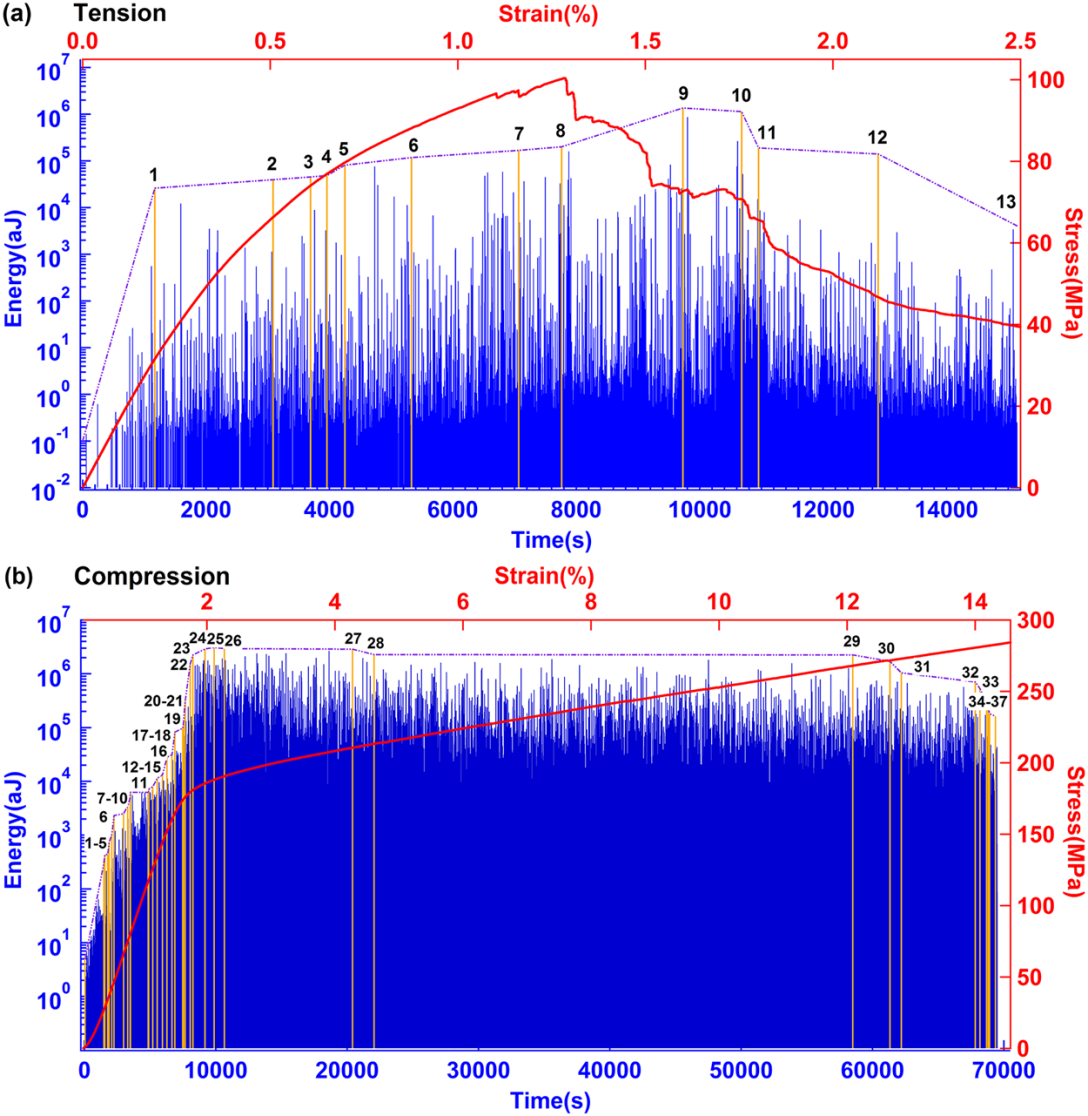
Stress-strain curve and time sequence of AE energy under tension (**a**) and compression (**b**).

Metallic alloys with the global composition of 25%Ho and 75%Mg were cast together. The porosity was ca. 11.7%. Individual grains were distributed fairly uniformly throughout the sample with holes and Ho inclusions (Fig. 1 and supplementary Fig. S1). Figure 2 shows the stress-strain curve of Mg-Ho alloy during tension and compression. The stress-strain curve under tension (Fig. 2a) undergoes elastic and yield/plastic deformation. Fracture starts at an engineering strain of 1.3%. The fracture process lasts for another 1.2% engineering strain where the sample does not completely break, indicating some ductility. The stress-strain curve under compression (Fig. 2b) shows elastic, yield, and plastic deformation, and then the sample breaks suddenly. The definition of elastic, yield, and plastic regime are given in refs^20,21^.

AE signals are emitted during tension and compression (Fig. 2). The experimental observation lasted until the final collapse (after ca. 20 hours for compression and ca. 4 hours for tension). The sequences are stationary for compression after an initial increase. The tension data remain non-stationary throughout the experiment. A super-jerk analysis^22–24^ was performed using super-jerks as shown by yellow AE signals in Fig. 2. The ranks of the super-jerk are indicated with 13 super-jerks in tension and 37 in compression. The probability distribution function of energy was calculated for each interval between super-jerks. We observed that some adjacent intervals had exactly the same power law exponents so that these intervals were merged for further analysis. In tension experiments, only two intervals show experimentally different exponents, namely those between super-jerks 1 and 8 (yield/plastic regime), and 8 and 13 (fracture regime). Under compression, super-jerks separate the AE signals into 12 groups. The groups are 1–5, 6, 7–10, 11 (elastic regime), 12–15, 16, 17–18, 19 (yield regime), 20–25, 26–31, 32–33 and 34–37 (plastic regime).

### Energy distribution of AE signals under tension and compression

**Figure 3:**
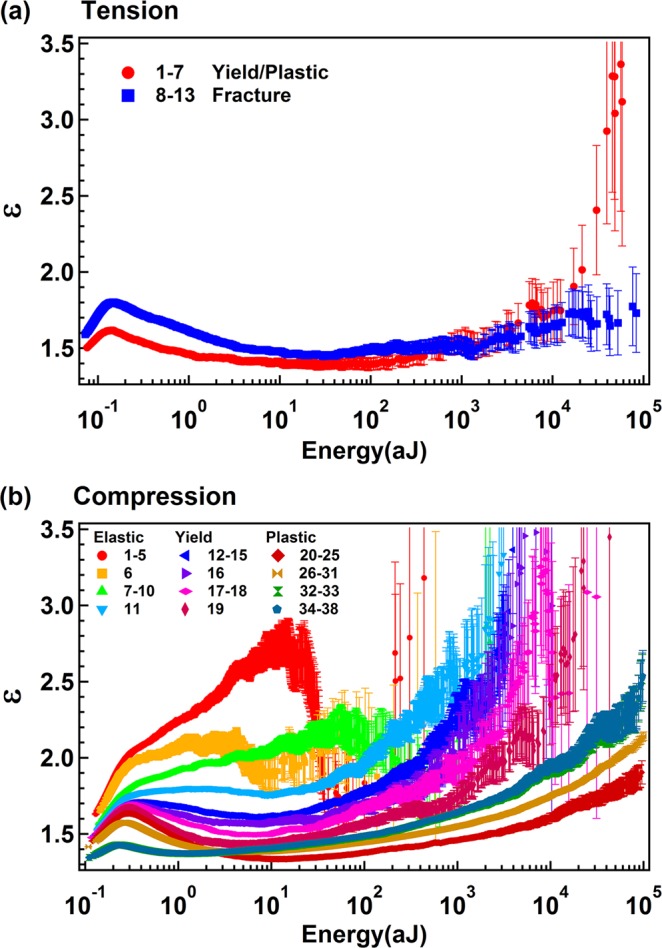
Representative Maximum Likelihood curves for tension (**a**) and compression (**b**).

**Figure 4:**
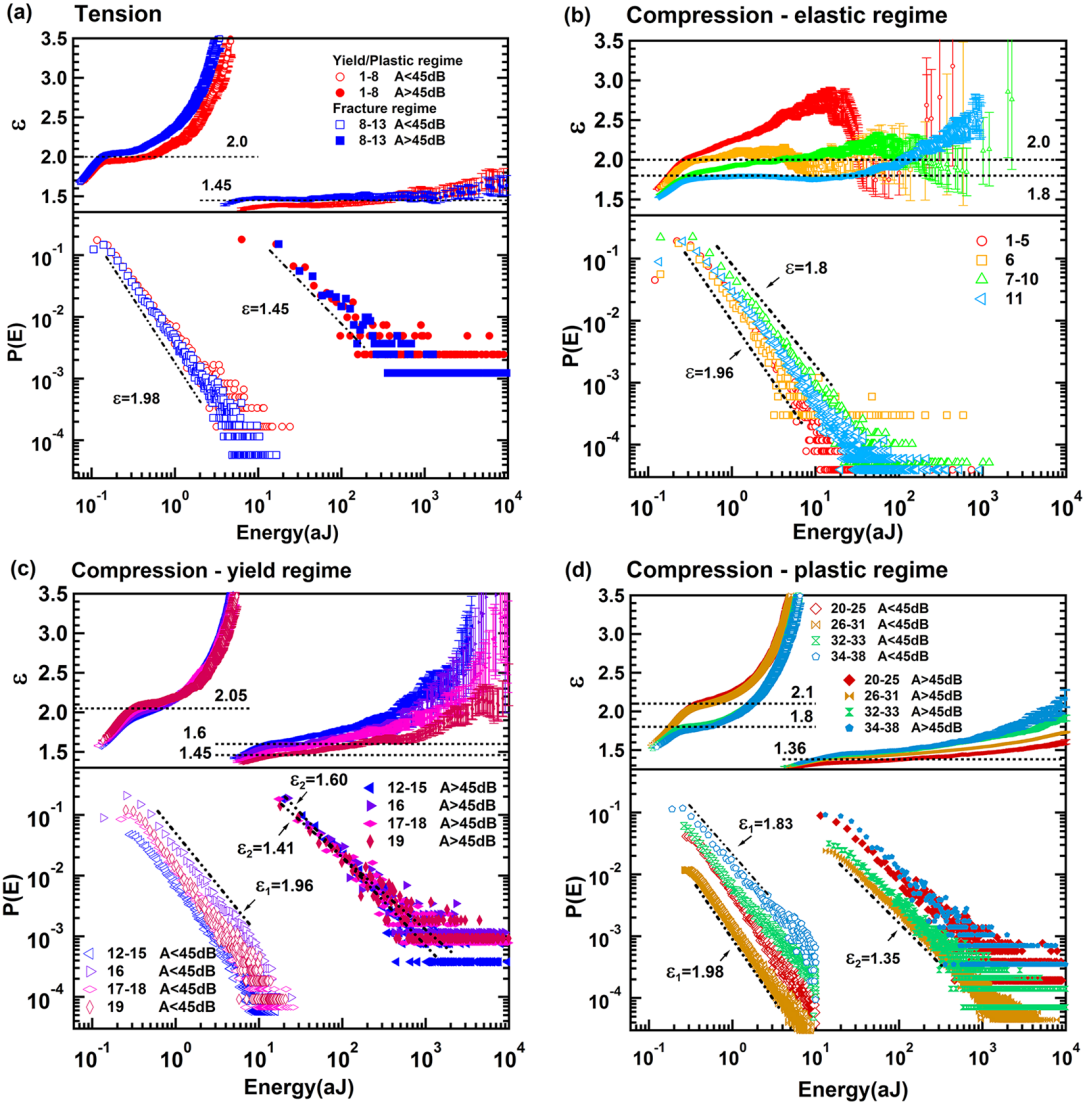
(**a**) Probability distribution function and maximum likelihood of AE energy under tension with two exponents ε_1_ = 1.98 ± 0.06 and ε_2_ = 1.45 ± 0.05. Probability distribution function and maximum likelihood of AE energy in compression test after separated into elastic (**b**), yield (**c**), and plastic regime (**d**). Super-jerk intervals show exponents 1.96 ± 0.04 and 1.8 ± 0.01 for elastic regime (**b**), mixing of two exponents ε_1_ = 1.96 ± 0.06 and ε_2_ = 1.41 ± 0.01 in the yield regime (**c**), and a mix of two exponents ε_1_ = 1.98 ± 0.01 and ε_2_ = 1.35 ± 0.04 in the plastic regime (**d**).

The jerks spectra were analysed using the Maximum Likelihood (ML) method^25,26^. The ML curves of AE signals under tension (Fig. 3a), after super-jerk separation, show a maximum near the onset of the ML plateau and then relax to a minimum with ε ~ 1.4. This profile is virtually identical with those predicted in ref.^16^ for scenarios where power law distributions were superimposed. We then analysed all super-jerk intervals and found two different energy exponents as function of the energy threshold of AE signals. Under tension, we find mixing between ‘strong’ and ‘weak’ AE signals with little dependence on the time evolution (Fig. 4a). An energy exponent near 1.96 is seen for all sequences with AE amplitudes <45 dB, and 1.45 for AE amplitudes >45 dB. This indicates that ‘strong’ and ‘weak’ energy signals follow different power law distributions (Fig. 4a). Each subsequence remains scale invariant with little visible cut-off effects. We note that the energy of “weak” AE signals (0.01 ~ 30 aJ) is 1–2 orders higher than the noise energy in Supplementary Fig. S2 (maximum value: 0.2 aJ) so that the “weak” signals are not related to background noise.

ML curves of AE signals under compression are shown in Fig. 3b. ML curves with super-jerk ranks <11 (elastic regime) show only one exponent ε ~ 1.96. The compression behavior then changes dramatically for super-jerks 12–19 (yield regime) when exponent mixing increases with ε ~ 2 and ε ~ 1.4. After super-jerk rank 20 (plastic regime) the spectrum becomes stationary (Fig. 2b) and mixing is always observed (Fig. 3b). We note that, in the yield regime there is no time evolution for weak signals with ε ~ 1.96, while big jerks evolve from an initial value of ε ~ 1.6 to ε ~ 1.41 at the end of the yield regime (Fig. 4c). In the plastic regime, the small jerks show an energy exponent 1.98, which reduces to 1.83 near the final collapse, while the high amplitude data show exponents near the mean field value 4/3 (Fig. 4d). In summary, values of ε near 1.96 are seen for all data in the elastic regime, and for all initial data in the yield and plastic regime for small jerks (only near the final collapse we find ε = 1.8 for weak jerks). All strong jerks show ε ~ 1.4, which is slightly above the mean field value 4/3.

### Inter-event times, omori scaling, and bath law

**Figure 5:**
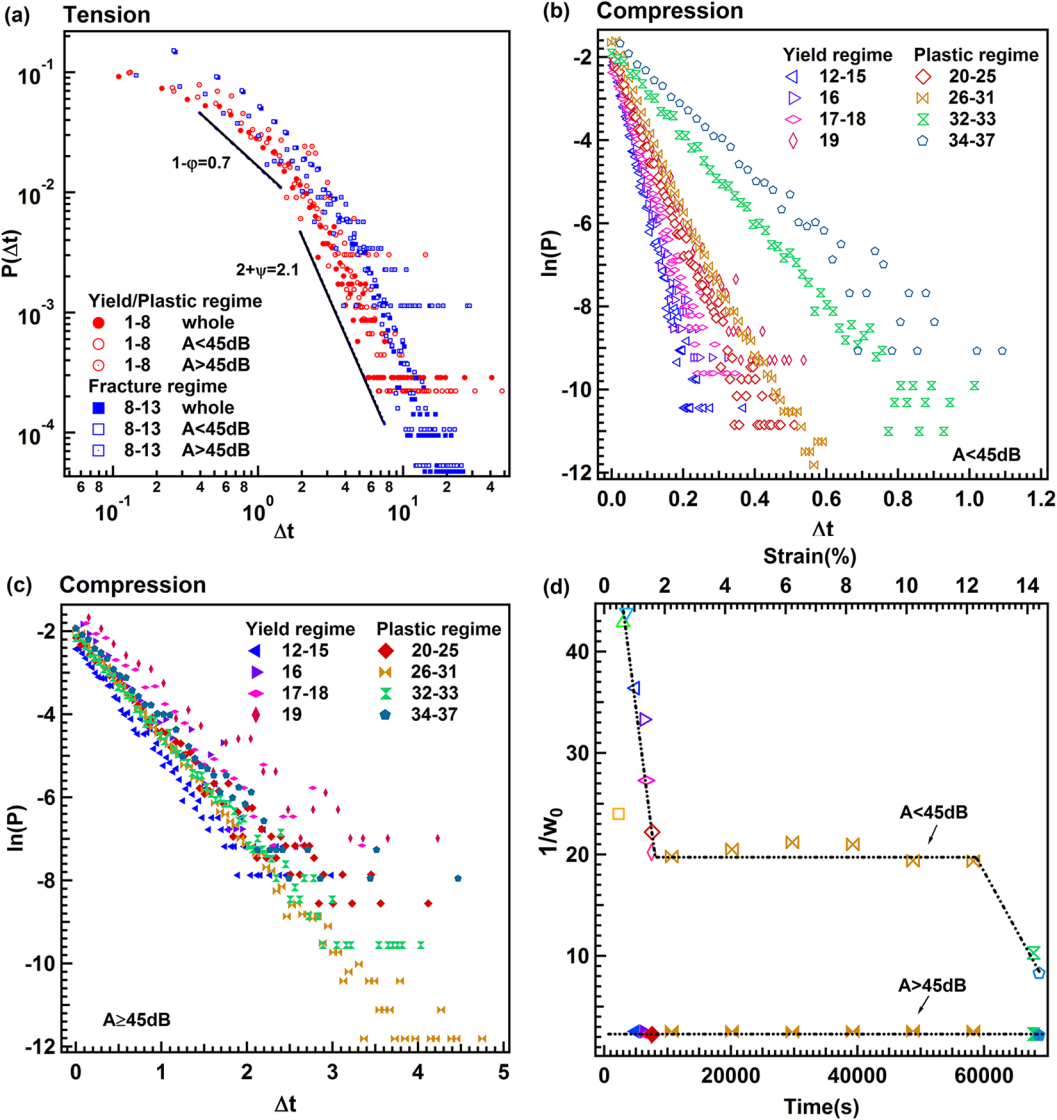
Distributions of inter-event times are power law distributed for tension (**a**) and exponential for compression (**b,c**). Evolution of 1/w_0_ of P(Δt) ~exp − (Δt/w_0_) with strain under compression shows in (**d**). The upper curve relates to the strong signal set, the lower curve to the weak signal set.

Time intervals between jerks (‘inter-event times’ or ‘waiting times’) were measured at small thresholds (~21.1 dB)^27,28^. Under tension, the probability distribution function, PDF, of waiting time shows two exponents for short and long waiting times, −0.7 and −2.1 (Fig. 5a), which is common for porous collapse avalanches^7,12–14^. In contrast, we do not observe power law distributions for inter-event times under compression (Fig. 5b,c). Instead, the inter-event times scale exponentially ~exp − (Δt/w_0_) with 1/w_0_ near 2.5 s^−1^ (strong AE signals) and 10 to 45 s^−1^ (weak AE signals, Fig. 5d). The weak signals show a decay in the initial plastic regime, a plateau between the yield regime near strain ~2% and a final collapse regime.

**Figure 6:**
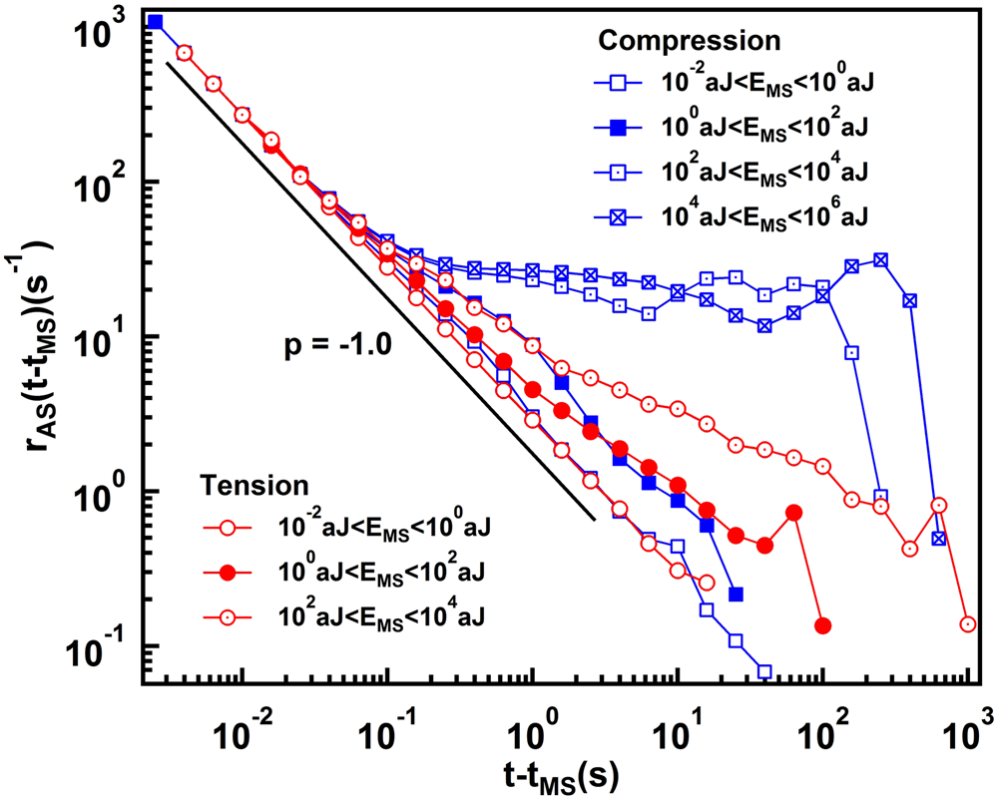
Number of aftershocks per unit time during tension and compression, r_AS_/(t − t_MS_), as function of the time distance from the main shock for different thresholds in each panel, as indicated by the legend. Main shocks are defined as the events in the energy range indicated by the legend. The black line indicates the Omori’s behavior with p = −1.0 ± 0.01.

The waiting time distribution is closely related to the number of aftershocks subsequent to mainshocks. The aftershock distribution is described by the Omori exponent p^29–32^. We noticed that the Omori law is fulfilled for all mainshock (MS) events with energies between 10^−2^ and 10^2^ aJ. The results in Fig. 6 show p ~ 1 for all the MS energies <100 aJ. Derivation from the Omori law are found for MS energies >100 aJ. These non-Omori behavior might be sufficient to render the inter-event distribution functions for these cases to exponential or Poisson distributions.

**Figure 7:**
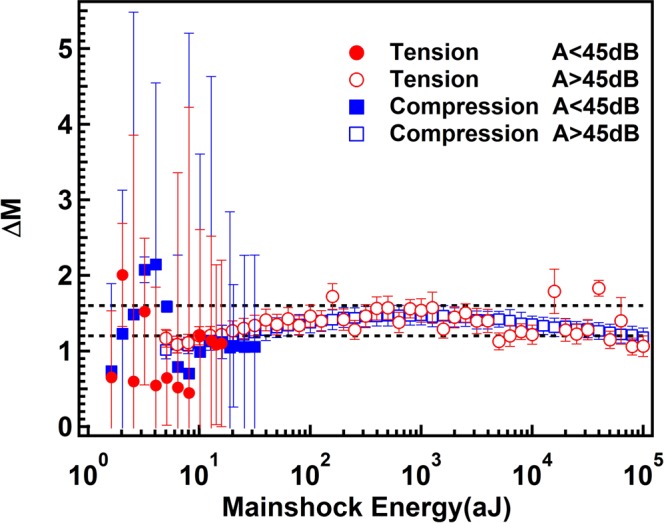
Bath’s law for strong >45 dB (open symbols) and weak AE signals amplitudes <45 dB (solid symbols). Strong amplitudes follow the Bath’s law with ΔM below 1.4. Weak signals show large fluctuations below 10 aJ.

We then plot the normalized ratio (ΔM) between the energy of the mainshock E_MS_ and its largest aftershock E_AS*_ as function of the mainshock energy E_MS_ in Fig. 7. Bath’s law^33,34^ states that ΔM = log (|E_MS_|/|E_AS*_|) ~ 1.2. For the strong AE signals, ΔM is indeed ~1.2, and small bending of the ΔM curves correlates with previous simulation results of theoretical models^34^. This is not the case for the weak AE signals where even significant averaging leads to excessive data scatter. While the data are in the range near 1.2, they do not display any Bath’s law as seen for strong AE signals. This may indicate that the aftershock sequences depend on the chemical composition of the grains in which the dislocations move.

### Exponent scaling

**Figure 8:**
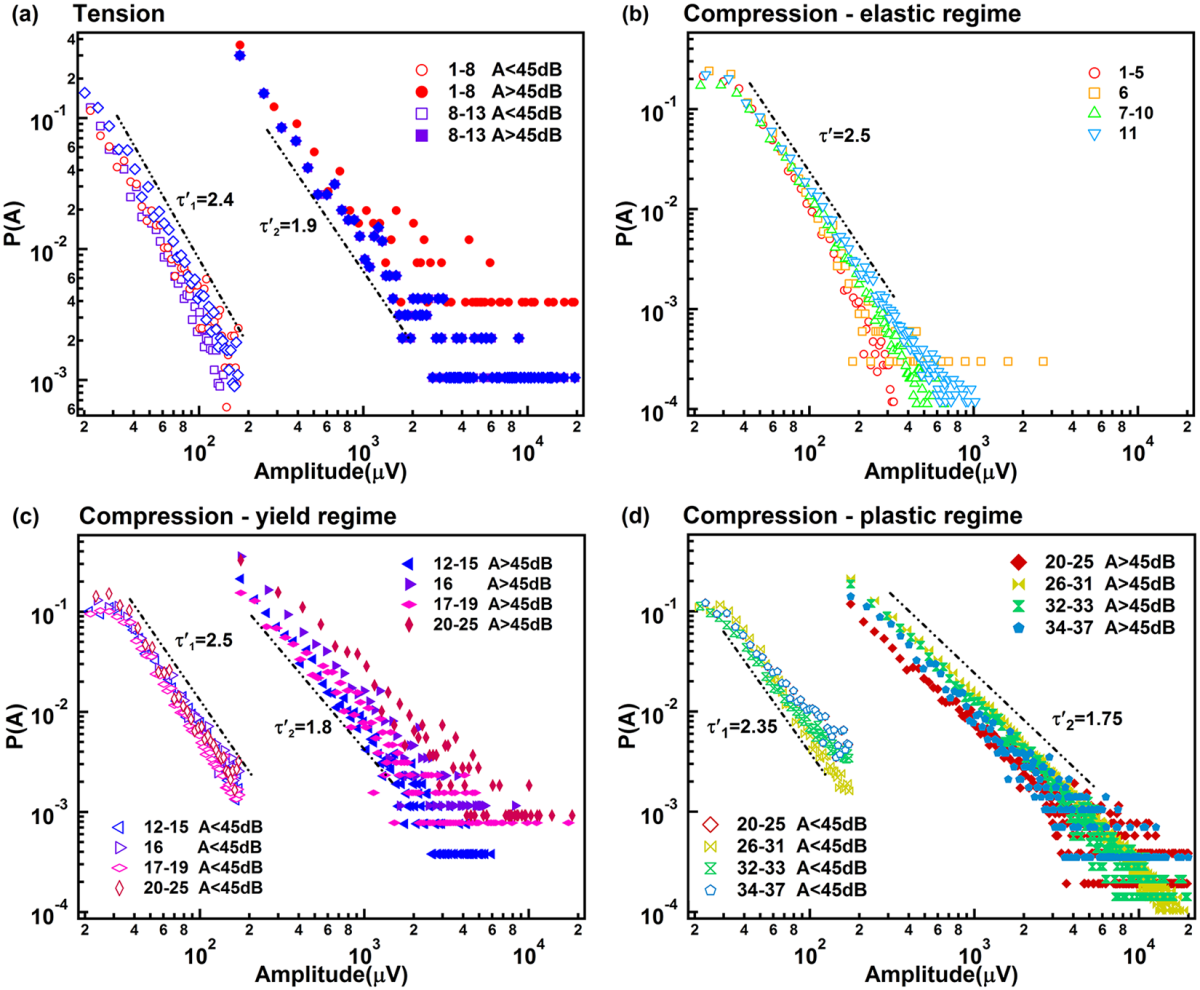
(**a**) Probability distribution function of AE amplitude in tension test, which show two exponents τ′_1_ = 2.4 ± 0.1 τ′_2_ = 1.9 ± 0.08. (**b–d**) Show probability distribution function of AE amplitude in compression test after separated into elastic, yield and plastic regime. Super-jerk intervals show exponents 2.5 for elastic region (**b**), mixing of two exponents τ′_1_ = 2.5 ± 0.05 and τ′_2_ = 1.8 ± 0.1 in the yield region (**c**), and a mix of two exponents τ′_1_ = 2.35 ± 0.06 and τ′_2_ = 1.75 ± 0.06 in the plastic region (**d**).

**Figure 9:**
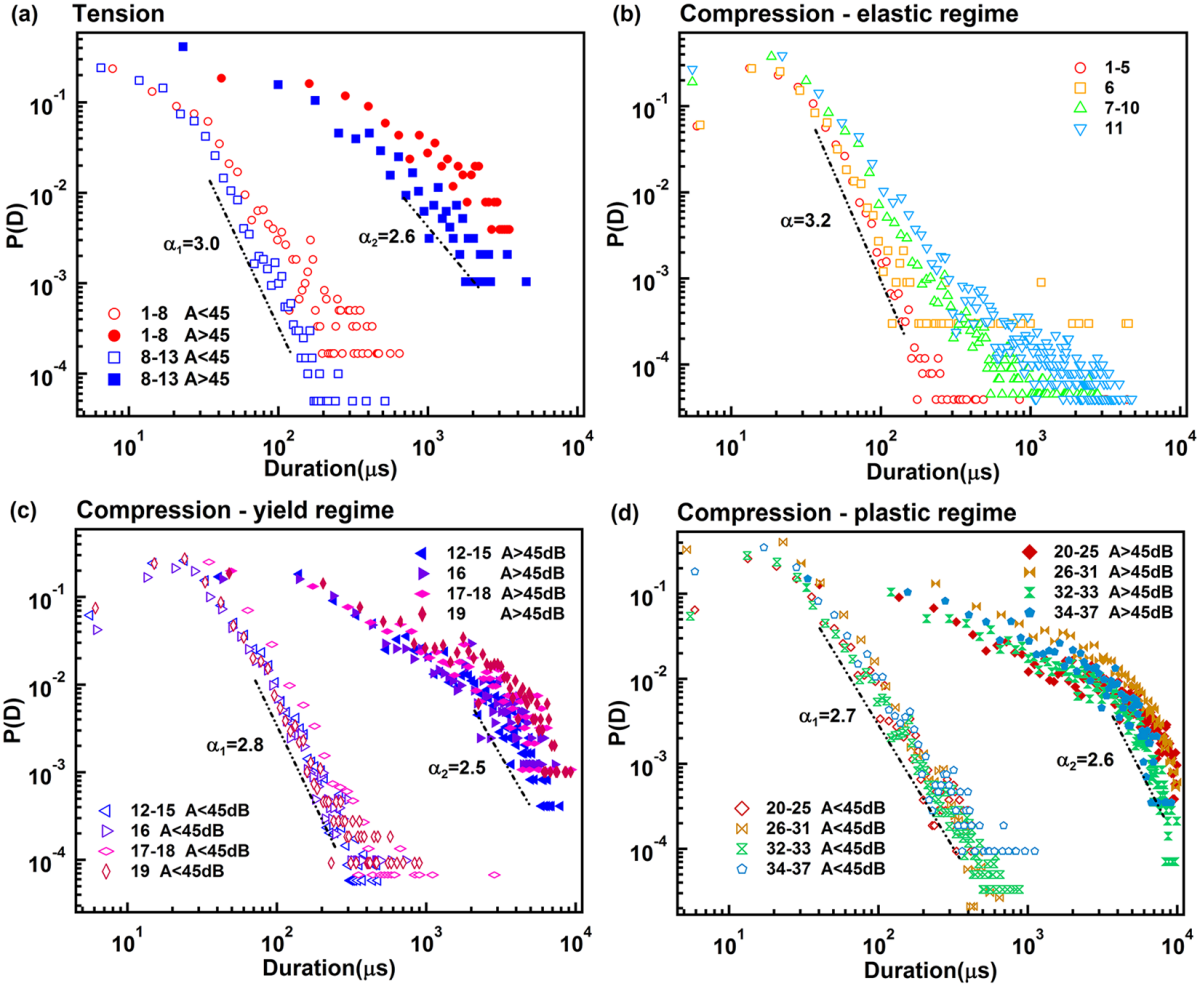
(**a**) Probability distribution function of duration in tension test, which show two exponents α_1_ = 3.0 ± 0.2 α_2_ = 2.6 ± 0.01. (**b–d**) Show probability distribution function of duration in compression test after separated into elastic, yield and plastic regime. Super-jerk intervals show exponents 3.2 for elastic region (**b**), mixing of two exponents α_1_ = 2.8 ± 0.3 and α_2_ = 2.5 ± 0.14 in the yield region (**c**), and a mix of two exponents α_1_ = 2.7 ± 0.22 and α_2_ = 2.6 ± 0.2 in the plastic region (**d**). The larger exponents are always for the weaker jerks, the smaller exponents for bigger jerks.

**Figure 10:**
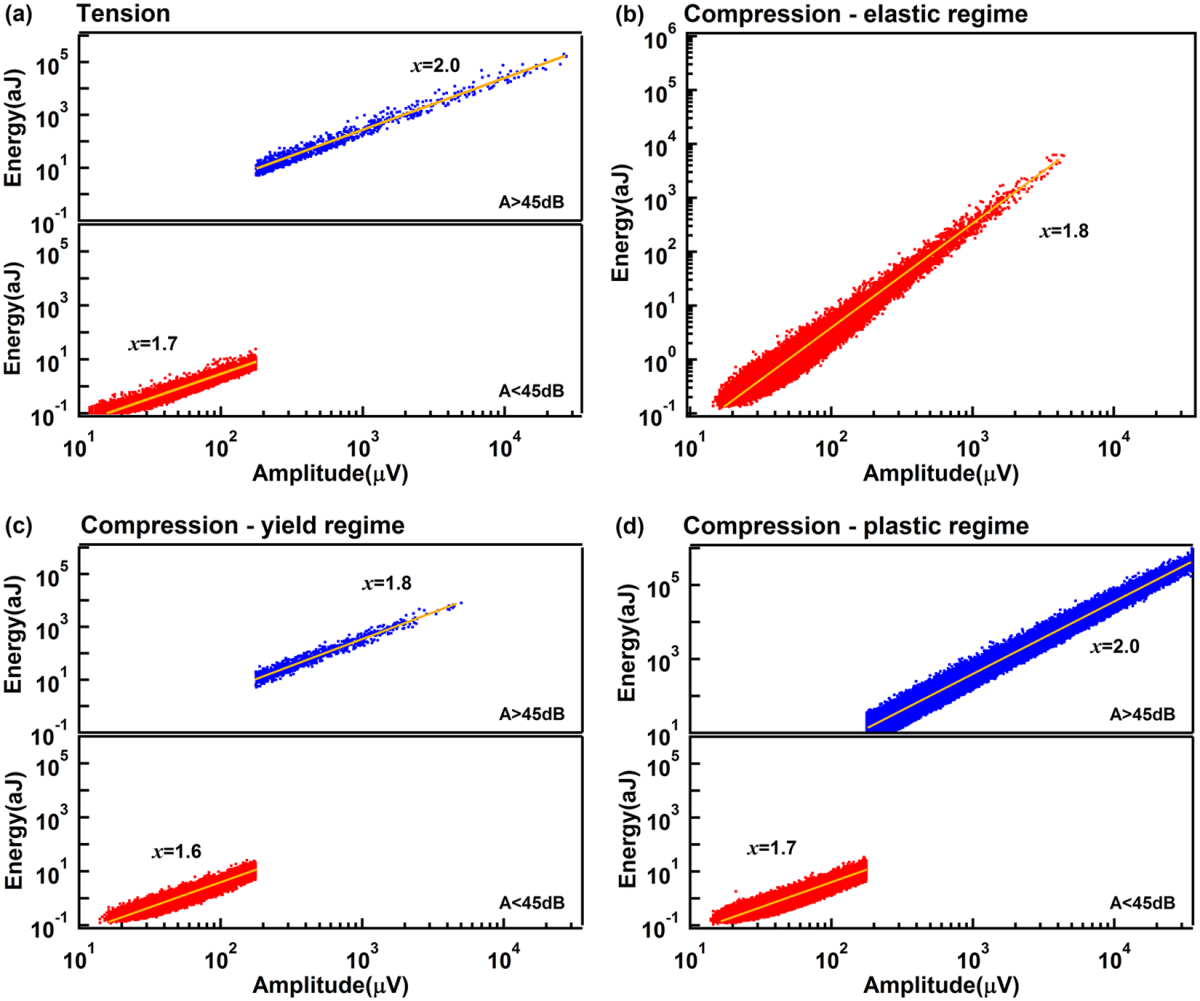
Correlation between the energies and the amplitudes of AE signals is power law distributed with E ~ A^*x*^ with *x* around 1.7 ± 0.1 for weak signals and 1.93 ± 0.1 for strong signals.

**Figure 11:**
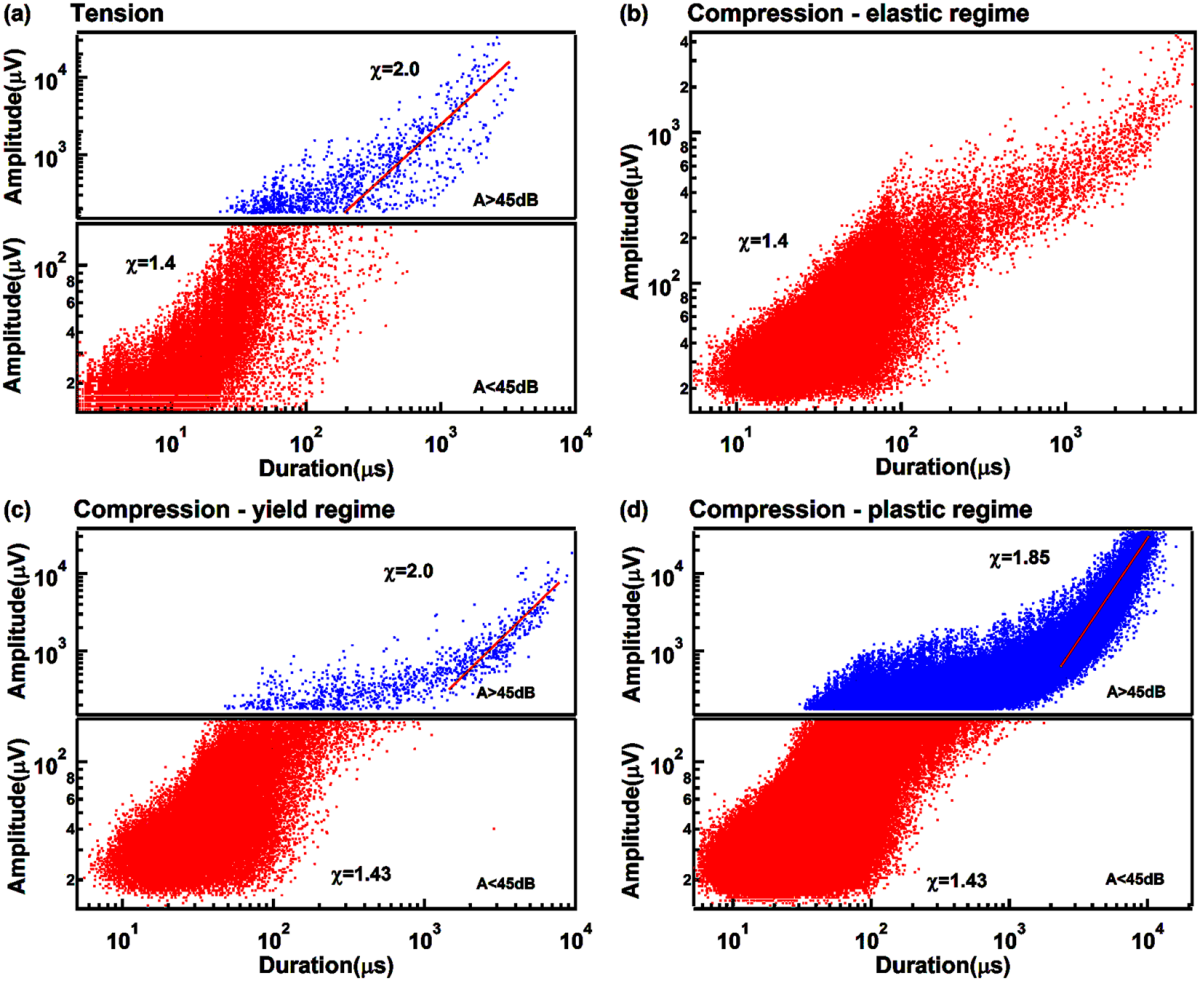
Amplitude versus duration frequency maps for tension (**a**) and compression (**b–d**). Which follow power law distribution A ~ T^χ^ with χ αround 1.42 ± 0.02 for weak signals and 1.95 ± 0.15 for strong signals.

The exponents of the power law distributions for the burst AE energy E, peak amplitude A, duration T and the correlations E(A), A(T) are shown in the Figs 8–11. Table 1 summarizes the exponents evaluated in this work where we follow the nomenclature^17^ and references in that paper. The exponents ε, τ′, α, *x*, χ, are linked by the assumption of having two power-law marginal probabilities from a bivariate density distribution. Theoretically, the relationship between these exponents is for the force integrated mean field model ${\rm{\tau }}^{\prime} -1=x(\varepsilon -1)=(\alpha -1)/\chi =1.33$, and ${\rm{\tau }}^{\prime} -1=x(\varepsilon -1)=(\alpha -1)/\chi =0.66$ for the mean field model.

**Table 1:** Exponents for power law distributions in Mg/Ho for strong and weak AE signals.

Parameter	MF value/Force integrated^17^	Experimental (weak/strong)
ε (Energy)	1.33/1.67	1.92 ± 0.12/1.40 ± 0.1
τ′ (Amplitude)	1.67/2.33	2.44 ± 0.09/1.82 ± 0.08
α (Duration)	2.0/3.0	3.0 ± 0.2/2.56 ± 0.2
*x* (Energy-Amplitude)	2.0/2.0	1.7 ± 0.1/1.93 ± 0.1
χ(Amplitude-Duration)	1.5/1.5	1.42 ± 0.02/1.95 ± 0.15

Interestingly, the comparison between experiment and theoretical model (Table 2) shows that the exponents of weak AE signals follow approximately the forced integrated mean filed model with $${\rm{\tau }}^{\prime} -1=(\alpha -1)/\chi \,\approx $$ $$x(\varepsilon -1)=$$ $$1.43\pm 0.15$$, while exponents of strong AE signals are nearer to the mean field model with $${\rm{\tau }}^{\prime} -1=$$$$(\alpha -1)/$$ $$\chi =x(\varepsilon -1)=0.8\pm 0.03$$.

**Table 2:** Exponents scaling relations of the power law distributions for energy E, amplitude A, duration T and the correlations E(A), A(T).

Signal type	correlation	Theoretical value MF/Force integrated^17^	Experimental value
Weak AE Signals	τ′ − 1	0.67/1.33	1.44 ± 0.09
	*x*(*ε* − 1)	0.66/1.34	1.56 ± 0.25
	(α − 1)/χ	0.67/1.33	1.41 ± 0.12
Strong AE Signals	τ′ − 1	0.67/1.33	0.82 ± 0.08
	*x*(*ε* − 1)	0.66/1.34	0.77 ± 0.25
	(α − 1)/χ	0.67/1.33	0.8 ± 0.18

## Discussion

Avalanches in heterogeneous Mg-Ho samples show power law distributed AE energies and occur predominantly in two AE energy windows. Signals with strength >45 dB dominate avalanches with energy exponents near 1.4 while amplitude of signals <45 dB dominate the exponent curves with ε = 1.96. Similar differences are found for the amplitude and duration distributions while other properties appear identical for both data sets. A typical example is the Omori law, which shows the same exponents for all signals smaller than 100 aJ. This reproduces previous results^7,35^ which show that the Omori scaling is virtually identical for a large variety of avalanche mechanisms ranging from porous collapse^7–12^, granular materials^36^ and moving twin boundaries^15,18,37–39^. The superposition of different avalanche mechanisms is hence not expected to modify these quantities.

Inter-event time distributions in tension experiments are power law distributed. In contrast, in compression experiments they differ greatly between the two mechanisms. Data show exponential distributions with two different time constants for small and large energy exponents. Weak AE signals, which constitute the majority of the data, show inter-event times with an approximately constant time scale w_o_ = 0.4 s. The strong events, on the other hand, show smaller time constants (0.1 s < w_o_ < 0.02 s), which increase from the initial compression data to a large plateau before increasing further when approaching the final collapse. These compression avalanches show hence a dependence on the time evolution, unlike the scale invariance in tension experiments, and they are different for the two types of avalanches. Inter-event time distributions for compression are not scale invariant, but display characteristic time scales for waiting times between avalanches.

We can now compare our results with previous measurements. The strong AE signals relate to porous collapse^7–12^ and reproduce the typical energy exponents for nano-porous vycor^7^ and and are slightly higher for macro porous goethite^11^, berlinite^12^, and alumina Al_2_O_3_^10^. These exponents are close to our value ε = 1.4. Porous collapse avalanches can extend over long duration times^18^, which agrees with our findings. Under compression, subsequent collapse events are highly correlated and the inter-event times are depending on the relaxation of the sample. This relaxation relates to local hardening as discussed by ref.^40^.

**Figure 12:**
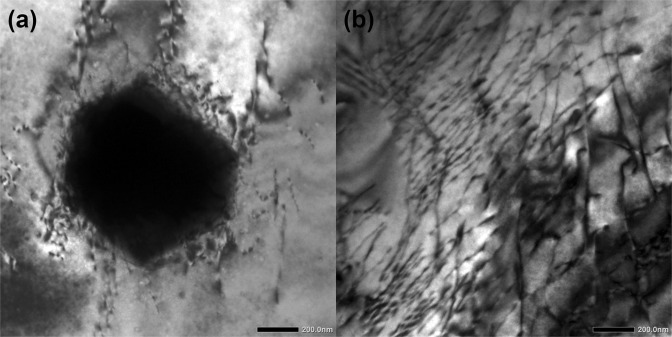
TEM figure shows Ho granule with surrounding dislocations (**a**) and dislocations in Mg matrix (**b**) in the sample after compression.

The second avalanche mechanism relates to dislocations. These events are much more numerous in our sample than porous collapses as we observe high dislocation densities but few holes in the samples (shown in Fig. 12). Dislocations commonly show large energy exponents near 1.9^15,41^, and amplitude exponents near 2.1^42,43^. These values are similar to our results (ε = 1.96, α = 2.2). The failure of the Bath’s law equally seems to support this identification as no Bath’s law has been reported in dislocation systems so far. There is a possibility that grain friction and grain micro fracture might also be involved in the weak AE signals. Nevertheless, considering the very large grain size (~0.5 mm) and relatively small size of sample cross-section (3 mm × 1 mm for tension and diameter = 6 mm for compression), only a few events from grain friction and grain micro fracture are likely to be monitored by AE.

The analysis shows clearly that the scale invariance of the two power laws (Figs 3 and 4) does not impede us from a detailed analysis of the two processes. Overlap between the spectra exists with some porous collapse signals for weak AE signals of the dislocation movements. There are many more dislocation signals so that the tail of the porous signals hardly matters for the low energy analysis. Inversely, the few high amplitude AE signals of the dislocations at the end of the power law have a very low probability and are swamped by strong AE signals related to the porous collapse.

In conclusion, we note that coinciding and competing avalanche mechanisms occur for many materials. Our study of Mg-Ho alloys with a complex but typical microstructure shows that investigations of mixed avalanches are possible using acoustic emission (AE) measurements. We expect that in future developments of AE facilities the investigations of several collapse mechanisms can be extended from heterogeneous to homogeneous materials.

## Methods

### Sample description

Averaged EDS spectra of Ho-rich and Mg-rich alloys (see Supplementary Fig. S1) show the local composition is for Mg rich parts: Ho 46 wt%, Mg 38 wt%, O 0.4 wt% and for the Ho-rich parts: Ho 77 wt%, Mg 10.3 wt%, O 1.3 wt%. The small difference from 100% may stem from surface roughness, porosity of the sample or from inherent problems in the PB-ZAF quantification method, such as the theoretical Ho spectrum used for the fitting procedure. Assuming oxygen is only present in the oxidized superficial layer and normalizing to 100, we obtain: 55 wt% Mg, 45 wt% Ho for a Ho-rich area; 88 wt% Mg 12 wt% Ho for a Mg-rich area. These volume percentages correspond to a composition of Mg_0.89_Ho_0.11_ in a Ho-rich and Mg_0.98_Ho_0.02_ for a Ho-poor parts of the sample.

### Experimental

Uniaxial tension and compression of porous Mg-Ho alloys were measured using an Instron 5969 Universal Testing Machine. The samples for tension experiments were dog-bone-shaped with a gauge range of 10 mm × 3 mm × 1 mm. The compression samples were cylinders with high *H* = 15 mm and diameter *φ* = 6 mm. Tension and compression experiments were performed at room temperature with velocity 10^−3^ mm/min.

A piezoelectric sensor (Vallen-Systeme GmbH) with a frequency band of 200–800 kHz was attached to the sample surface. AE signals were first pre-amplified by 43 dB and then transferred to the AMSY-6 AE-measurement system (Vallen-Systeme GmbH) using a frequency range 95–850 kHz. The amplitude *A* is recorded in dB which follows the expression dB = [20 Log (|*V*_*sensor*_|*/*1 *μ*V)], where *V*_*sensor*_ is the peak voltage output by sensor, and the brackets round the value to its nearest integer in dB.

An AE signal is defined as a ‘burst’ in the noise spectrum. The start of a burst is determined by a first threshold crossing, and ended at second threshold crossing or when Duration Discrimination time (DDT, defines a time period in which no threshold crossing must occur in order that an end of hit is determined) expired without any threshold crossing. A threshold of 21.1 dB was determined by prior rubber experiments to evaluate the internal noise of the experimental arrangement. We changed the DDT from 50 μs to 1000 μs, and found that DDT ~ 300 μs fits the porous collapse well, while DDT ~ 50 μs fits the dislocation movements well. In order to capture both porous collapse and dislocation movements, the value of DDT = 100 μs was chosen. The AE energy is the integral of the square of the output voltage over the burst interval. The energy is then electronically calibrated into atto Joules, aJ.

In order to ensure that all the AE signals collected during the tension/compression experiment stem from the sample, we performed noise separation measurement. We first investigated the noise of the full experiment using a dummy rubber sample with the same detector configuration as in the alloy experiment (see Supplementary Fig. S2). We also measured the noise of the instrument by attaching the detector to the sample grips. The distribution of background noise follows approximately a log normal distribution (see Supplementary Fig. S3) while the maximum noise energy is less than 0.2 aJ.

## Supplementary information

Supplementary Information
