# Supplementary material for: Acoustic Emission from Porous Collapse and Moving Dislocations in Granular Mg-Ho Alloys under Compression and Tension
Source: Sci Rep. 2019 Feb 4;9:1330. doi: 10.1038/s41598-018-37604-5 (PMC6361990; doi:10.1038/s41598-018-37604-5)
Supplement: Supplementary file 1 — Supplementary Information [file 41598_2018_37604_MOESM1_ESM.docx]

**Supplementary Information**

## Acoustic Emission from Porous Collapse and Moving Dislocations in Granular Mg-Ho Alloys under Compression and Tension

Yan Chen^1^, Xiangdong Ding^1,a^, Daqing Fang^1^, Jun Sun^1^ & Ekhard K.H.Salje^1,2,a^

1 State Key Laboratory for Mechanical Behavior of Materials, Xi’an Jiaotong University, Xi’an 710049, China

2 Department of Earth Sciences, University of Cambridge, Cambridge CB2 3EQ,

England

^a)^ E-mail addresses: ekhard@esc.cam.ac.uk (Ekhard K. H. Salje)

dingxd@mail.xjtu.edu.cn (Xiangdong Ding)

Experimental details

**
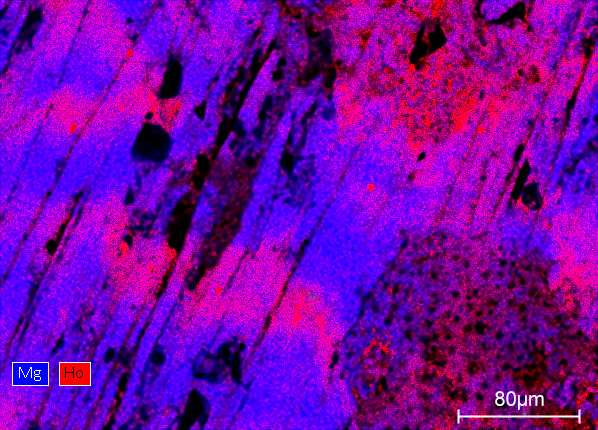
**

**Figure S****1.** SEM image of the composition distribution in as-cast Mg-Ho alloy with 11.7% porosity.

**
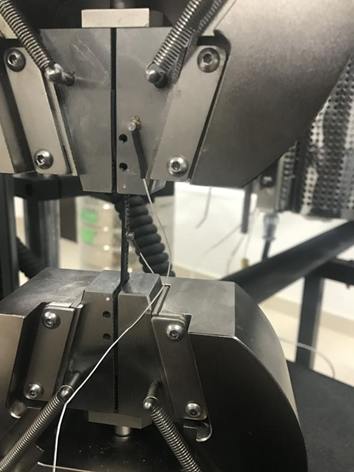
**

**Figure S2.** (a) Background noise experiment for tension and compression(b)

**
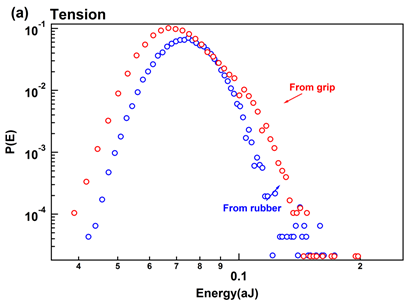

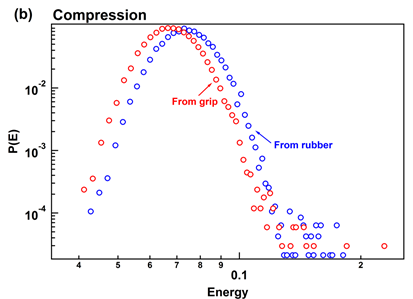
**

**Figure S3.** (a) Distribution of background noise during tension and compression (b)
